# Protozoan and Helminth Contamination in Ecuadorian Agricultural Products: Dominance of *Blastocystis* sp.

**DOI:** 10.1155/japr/4620160

**Published:** 2025-03-03

**Authors:** Luisa Carolina González-Ramírez, Cristian Andrés Quito López, Verónica Carolin Rojano Silva, Ximena del Rocío Robalino Flores, Silvia Paola Monar Basantes, José G. Prato, César Díaz-Godínez, Julio César Carrero

**Affiliations:** ^1^Grupo de Investigación “Análisis de Muestras Biológicas y Forenses”, Laboratorio Clínico, Facultad de Ciencias de la Salud, Universidad Nacional de Chimborazo, Riobamba, Ecuador; ^2^Semillero de Investigación “Parásitos”, Laboratorio Clínico, Facultad de Ciencias de la Salud, Universidad Nacional de Chimborazo, Riobamba, Ecuador; ^3^Grupo de Investigación Estudios Interdisciplinarios, Facultad de Ingeniería, Universidad Nacional de Chimborazo, Riobamba, Ecuador; ^4^Departamento de Inmunología, Instituto de Investigaciones Biomédicas, Universidad Nacional Autónoma de México, Mexico City, Mexico

**Keywords:** *Blastocystis* sp., contamination, Ecuador, food-borne diseases, fruits, intestinal parasites, leafy greens, vegetables

## Abstract

Foodborne diseases, particularly those caused by parasitic infections, pose significant public health challenges globally, particularly affecting vulnerable populations such as children. In this study, we investigated the parasitic contamination in 773 samples of fruits, vegetables, and leafy greens from Ecuador's Chimborazo Province. Utilizing a cross-sectional, observational approach, samples were collected from the capital of the Guano canton, Chimborazo Province. The analysis revealed an alarming overall contamination frequency of 74.5%, with leafy greens exhibiting the highest contamination (238/275 [86.5%]), followed by vegetables (130/188 [69.1%]) and fruits (208/310 [67.1%]) (*X*^2^ = 32.793; *p* < 0.0001). Protozoa (71.8%) were four times more prevalent than helminths (16.9%) (*X*^2^ = 471.106; *p* < 0.0001), showing a higher frequency in leafy greens (228/275 [82.9%]) compared to fruits (205/310 [66.1%]) and vegetables (122/188 [64.9%]) (*X*^2^ = 26.113; *p* < 0.0001). In contrast, helminths were more frequently found in vegetables (54/188 [28.7%]) than in leafy greens (49/275 [17.8%]) and fruits (28/310 [9%]) (*X*^2^ = 32.469; *p* < 0.0001). Notably, *Blastocystis* sp. was the most frequently identified parasite in all types of produce, particularly in fruits (43.5%) and leafy greens (40.7%). These findings underscore the critical need to improve food safety protocols and sanitation practices in agricultural settings to mitigate health risks associated with parasitic infections, especially among children in socioeconomically disadvantaged rural communities.

## 1. Introduction

Foodborne diseases constitute a major global health issue. Among these, parasitic infections stand out because they are primarily transmitted through fecal contamination of water, soil, and agricultural products, which serve as important vehicles for the spread of infectious parasitic forms [1, 2]. Ingestion of leafy greens, vegetables, and fruits contaminated with protozoan cysts or oocysts and helminth eggs can lead to serious health consequences [2]. When ingested, enteric parasites cause digestive disorders such as diarrhea, vomiting, and abdominal pain, which can lead to dehydration or nutrient depletion. Chronic or severe infections may result in complications, including malnutrition and, in some cases, death [3, 4]. The large number of foodborne parasite species and high contamination rates underscore the need for robust surveillance and strict food safety regulations to prevent outbreaks, particularly in resource-limited regions [2].

According to the World Health Organization (WHO), nearly one-third of foodborne illness deaths worldwide occur in children under 5 years old. Its records indicate that Africa poses the largest burden of foodborne diseases per capita, with over 91 million people falling ill annually, while the Americas, with 77 million people, rank third [4]. Recent studies highlight significant global concerns regarding parasitic contamination in agricultural products, despite varying sanitation standards. Badri et al., through a meta-analysis of related publications, estimated the global prevalence of intestinal protozoan parasite contamination to be around 20% for vegetables and 13% for fruits. The most prevalent protozoan parasite in vegetables was *Cryptosporidium* spp. with 11%, while *Entamoeba histolytica* was the most common agent found in fruits with 9%. As expected, unwashed samples had the highest pooled prevalence of contamination with 22% [5]. On the other hand, Eslahi et al. performed a similar meta-analysis for helminth frequency, estimating a global incidence of 31% in vegetables and 20% in fruits. The highest incidence rate was found in the Western Pacific WHO region with 54%. The most prevalent helminths were *Ascaris lumbricoides* eggs and *Strongyloides stercoralis* larvae in 12% of vegetables and *S. stercoralis* larvae, *Toxocara* spp. eggs, and *Trichuris trichiura* eggs in 9% of fruits [6].

Contamination of vegetables and fruits has been identified throughout the world. In Europe, parasitic contamination has been reported in prepackaged salads from Italy, identifying *Cyclospora cayetanensis*, *Cryptosporidium* spp., *Toxoplasma gondii*, *Giardia duodenalis*, *Blastocystis hominis*, *Dientamoeba fragilis* [7], and *Echinococcus multilocularis* [8], as well as *Cryptosporidium* spp., *G. duodenalis*, and *E. histolytica* in berry fruits [9]. In Spain, high rates of parasitic contamination have been recorded in lettuce, where *G. duodenalis*, *Cryptosporidium* spp., *Acanthamoeba*, *T. gondii*, *Vermamoeba vermiformis*, *C. cayetanensis*, and *Blastocystis* sp. have been detected [10, 11]. In England, *Toxocara canis* and *Toxocara cati* have been reported in 25% of spinach samples [12]. In Asia, up to 63% of lettuce, cabbage, and basil from Indonesia were identified as contaminated with hookworm and *S. stercoralis* [13], while in Palestine, up to 47.5% contamination of zucchini was reported [14]. Africa also faces similar contamination rates, reaching 62.3% in vegetables and 60% in leafy greens [15]. In Latin America, on the other hand, surveys carried out in Argentina showed high rates of contamination of vegetables (58.6%) with *Cryptosporidium* sp. and hookworm [16], while studies in Brazil reported 70% contamination of fruits such as guava and grape [17] and in Colombia up to 100% contamination of lettuce with *Entamoeba* sp. and *S. stercoralis* [18]. In contrast, lower rates of contamination have been reported in other countries, such as 20% in carrots from Cuba [19] and 18.4% in onions and coriander from Peru [20].

There are few reports on the contamination of vegetables in Ecuador. The first study carried out in the coastal region of Manabí indicated a contamination rate in lettuce of 82.3%, of which 35.5% was with *Endolimax nana*; 8.1% with *Entamoeba* sp., *Giardia* sp., and *Chilomastix mesnili* complex; 6.5% with *Entamoeba coli* and *Blastocystis* spp.; 4.8% with *Balantidium coli*; 3.2% with adult nematodes; and 1.61% with *Pentatrichomonas hominis*, larvae of nematodes, and hookworms [21]. A study carried out by our group on agricultural products cultivated in the San Andrés parish of Guano canton in the Andean Province of Chimborazo showed a total contamination rate of 63.4%, with leafy greens showing the highest level of contamination at 76.9%, followed by vegetables at 67.8% and fruits at 48.4%. Contamination with protozoa was more prevalent (49.6%) than with helminths (15.5%), with *Blastocystis* sp. being the predominant protozoan (33.5%) [2]. Since the vegetables and fruits harvested in this region supply the other provinces of Ecuador and its capital, Quito, constant monitoring is a fundamental strategy to mitigate the risks associated with agricultural food consumption. The objective of this study was to evaluate the frequency of contamination with enteroparasites of leafy greens, vegetables, and fruits harvested in the capital of the Guano canton and to reveal their role as transmission vehicles of infectious parasitic forms in the region.

## 2. Methods and Materials

### 2.1. Study Area

**Figure 1:**
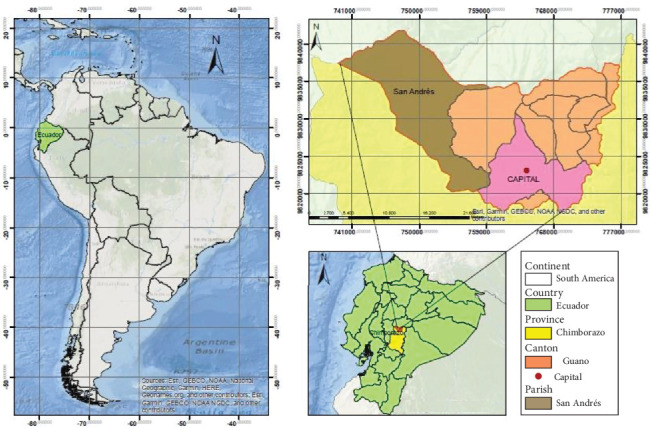
Geographical map of the study area.

The research was conducted in the capital of the Guano canton, province of Chimborazo (Figure 1), located at an altitude of 3900 m above sea level. The local temperature ranges between 5°C and 18°C, with annual precipitation varying between 500 and 1000 mm. There are two rainy seasons, from February to May and from October to November, with moderate rainfall in the remaining months during transition periods. Evapotranspiration contributes to soil dryness, caused by volcanic ash of variable textures, predominantly shallow loams with a pH ranging from 4.5 to 6.5.

### 2.2. Research Design

A cross-sectional, observational, and descriptive field study was conducted. The snowball sampling technique was applied, wherein a producer helped locate the nearest farm, and so on. All farms found were included in the sampling. The inclusion criterion required that all agricultural products be from fields in the capital of Guano canton. Products not cultivated in this locality were excluded.

### 2.3. Sampling

Farmers harvested leafy greens, vegetables, and fruits as they normally do in their fields. The samples were placed in plastic bags with hermetic seals. After being coded, they were immediately transported to the Research and Outreach Laboratory of the Faculty of Health Sciences of the University of Chimborazo, where they were processed and examined.

A total of 773 samples were analyzed, including 310 fruits of seven types: 50 strawberries (*Fragaria ananassa*), 50 blackberries (*Rubus glaucus*), 50 cape gooseberries (*Physalis peruviana*), 50 peaches (*Prunus persica*), 40 lemons (*Citrus limon*), 40 guavas (*Psidium guajava*), and 30 figs (*Ficus carica);* 188 vegetables of six types: 30 carrots (*Daucus carota*), 25 radishes (*Raphanus sativus*), 34 red onions (*Allium cepa* var. *rosum*), 34 white onions (*Allium strain* L), 25 lupin beans (*Lupinus mutabilis*), and 40 chili peppers (*Capsicum frutescens*); and 275 samples of leafy greens of eight types: 50 lettuces (*Lactuca sativa*), 30 celery (*Apium graveolens*), 30 parsley (*Petroselinum crispum*), 40 coriander (*Coriandrum sativum*), 40 watercress (*Nasturtium officinale*), 40 alfalfa (*Medicago sativa*), 20 cabbages (*Brassica oleracea*), and 25 chards (*Beta vulgaris*).

### 2.4. Ethical Considerations

The sampling was carried out with the appropriate authorization of the cantonal and parish decentralized autonomous governments. All farmers collected samples of their own crops (as they always do), knowing that the study benefits the community, without compromising the health of the population with respect to bioethical principles.

### 2.5. Parasitological Analysis

The protocol for the parasitological analysis was carried out according to Rivero de Rodríguez et al. [22]. An amount of 75 g of each sample of fruits, vegetables, or green leaves was added to 500 mL of previously filtered and boiled water. The content was mixed with a magnetic stirrer for 1 h, the plant remains were removed, and the solution was allowed to stand for 24 h. Subsequently, the solution was decanted, and the first fraction was collected in 15 mL tubes and subjected to centrifugation for 5 min at 800 × *g*. The supernatant was discarded, and the pellet was reconstituted in 400 *μ*L of physiological saline (0.85% NaCl). Each sample was observed under an optical microscope (Nikon E200) using 10X and 40X magnifications. Iodine solution and an ocular micrometer were used when necessary to stain the parasitic structures or measure their dimensions for identification. Additionally, a smear was prepared using a drop of the sediment and subjected to acid-fast staining (modified Ziehl–Neelsen technique) for the detection and identification of coccidia oocysts (*Cryptosporidium* or *Cyclospora*) at 100X magnification [23].

### 2.6. Statistical Analysis

The database was analyzed using SPSS Statistic 26.0 software (IBM). The difference in parasitic contamination between the various categories of plant products and the predominant parasite type in each plant species were compared using Pearson's chi-square test (*X*^2^) and Fisher's exact test when appropriate. A *p* value < 0.05 was considered statistically significant.

## 3. Results

### 3.1. Parasitic Contamination in the Agricultural Products

The analysis revealed an alarming overall contamination of 74.5%, with leafy greens exhibiting the highest frequency (238/275 [86.5%]), followed by vegetables (130/188 [69.1%]) and fruits (208/310 [67.1%]) (*X*^2^ = 32.793; *p* < 0.0001). The occurrence of protozoa was different in the three products analyzed: leafy greens (228/275 [82.9%]) (*X*^2^ = 233.038; *p* < 0.0001), fruits (205/310 [66.1%]) (*X*^2^ = 234.953; *p* < 0.0001), and vegetables (122/188 [64.9%]) (*X*^2^ = 49.393; *p* < 0.0001). The difference between groups was attributed to the higher frequencies of some protozoa in leafy greens, specifically *Eimeria* (85/275 [30.9%]) (*X*^2^ = 14.940; *p* = 0.0006), *Entamoeba* (51/275 [18.5%]) (*X*^2^ = 27.749; *p* < 0.0001), and *Balantidium* (19/275 [6.9%]) (*X*^2^ = 31.681; *p* < 0.001), as the other protozoan parasites did not show significant differences in prevalence among the three food types.

Protozoa found in 555/773 samples (71.8%) were four times more frequent than helminths found in 131/773 (16.9%) (*X*^2^ = 471.106; *p* < 0.0001) (Table 1). A higher occurrence of *Blastocystis* (314/773 [40.6%]) was observed when comparing protozoa (*X*^2^ = 1371.919; *p* < 0.0001). The prevalence of *Blastocystis* sp. among the analyzed product types exhibited slight percentage differences: fruits showed the highest contamination (43.5%), followed by leafy greens (40.7%) and vegetables (35.6%). However, no statistically significant differences were observed (*X*^2^ = 3.038; *p* = 0.2190) (Table 1).

In contrast, helminths were more frequently found in vegetables (54/188 [28.7%]) than in leafy greens (49/275 [17.8%]) and fruits (28/310 [9%]) (*X*^2^ = 32.469; *p* < 0.0001). Statistical analysis of helminth occurrence showed the highest contamination by *Strongylida* (16.3%) (*X*^2^ = 246.813; *p* < 0.0001). Details are in Table 1.

### 3.2. Occurrence of Parasites in Fruits

**Figure 2:**
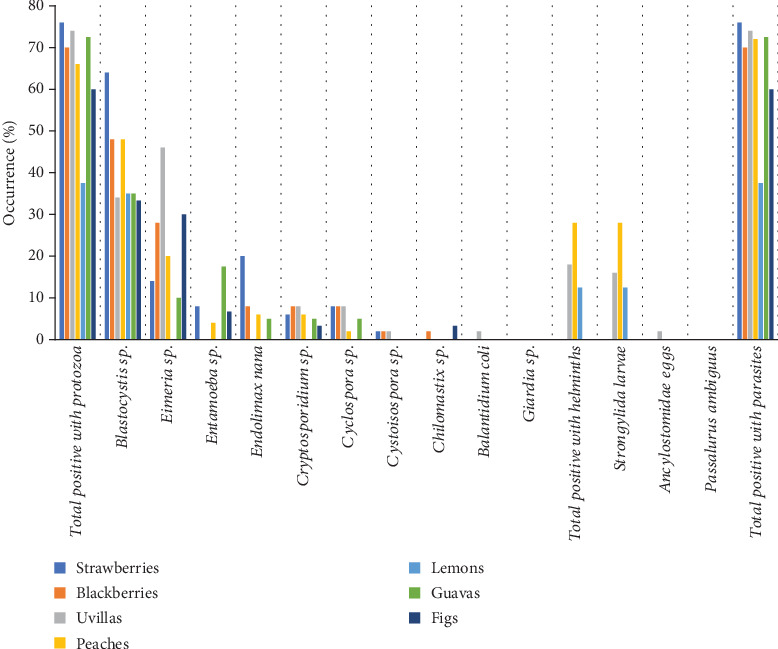
Parasitic contamination of fruits harvested in the capital of Guano canton.

Figure 2 shows the results of the incidence of the different types of parasites identified in the sampled fruits. It is observed that 67.1% of the samples analyzed presented parasitic contamination (Table 1), with strawberries presenting the highest frequency (38/50 [76%]), followed by frequencies greater than 60% for blackberries, cape gooseberries, peaches, guavas, and figs. Lemons showed the lowest frequency with 15/40 (37.5%). The protozoan most frequently detected in all types of fruits was *Blastocystis* sp. (135/310 [43.6%]), followed by *Eimeria* sp. (67/310 [21.6%]), which was found in all fruits except lemons. *E. nana* and *Cryptosporidium* sp. were found in 6.1% and 5.5% of the fruits, respectively. The other protozoa contaminated less than 5% of fruits, with *Giardia* sp. being notable for its absence in all analyzed fruits. Helminths were also absent in most of the analyzed fruit samples; only *Strongylida* larvae were detected with frequencies of 27/310 (8.7%) in peaches, 8/50 (16%) in cape gooseberries, and 5/40 (12.5%) in lemons.

### 3.3. Occurrence of Parasites in Vegetables

**Figure 3:**
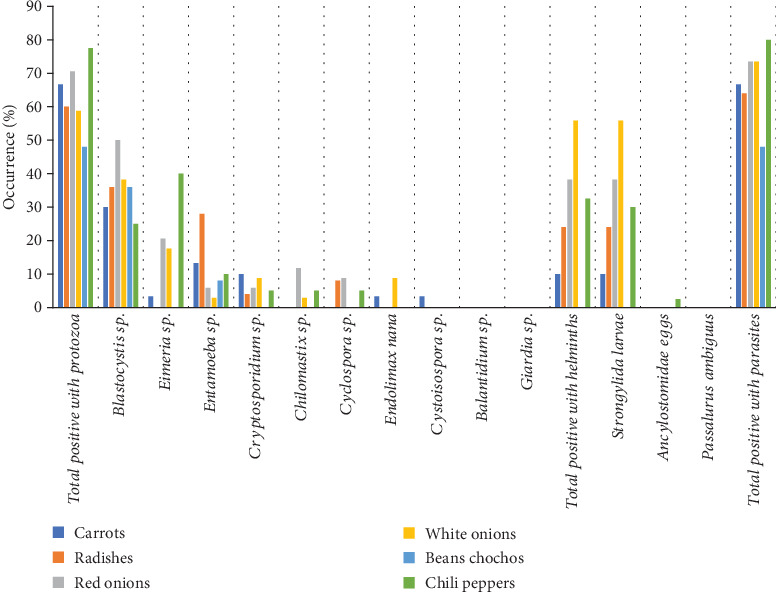
Parasitic contamination of vegetables harvested in the capital of Guano canton.

In this case, contaminating parasites were observed in 69.1% of the total samples analyzed (Table 1 and Figure 3). Chili peppers showed the highest prevalence of parasites (32/40 [80%]), followed by white and red onions (25/34 [73.5% each]), carrots (20/30 [66.7%]), radishes (16/25 [64%]), and, finally, lupin beans (32/40 [48%]). The most prevalent protozoa found were *Blastocystis* sp. (35.6%) in all types of vegetables; *Eimeria* sp. (16%), which was absent in radishes and lupin beans; and *Entamoeba* sp. (10.6%), also present in all vegetables. The remaining protozoa showed a prevalence below 10%. Notably, *Giardia* sp. and *Balantidium* sp. were absent. Regarding helminths, only *Strongylida* larvae were found in 28.7% of vegetables, threefold higher than in fruits (9.03%). The highest helminth contamination was observed in white onions (55.9%), and no contamination was found in lupin beans.

### 3.4. Occurrence of Parasites in Leafy Greens

**Figure 4:**
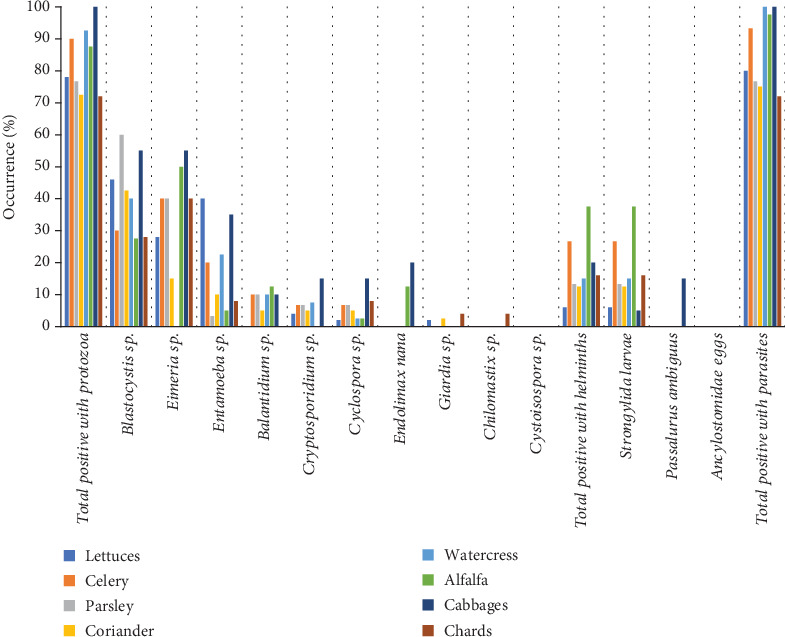
Parasitic contamination of leafy greens harvested in the capital of Guano canton.

Leafy greens exhibited the highest parasitic contamination (238/275 [86.5%]). All watercress and cabbage samples were contaminated with at least one of the target parasites (40/40 and 20/20, respectively). Protozoa were detected in 82.9% of the samples, with the highest percentages observed for *Blastocystis* sp. (40.7%), *Eimeria* sp. (30.9%), and *Entamoeba* sp. (18.5%). *Blastocystis*, *Entamoeba*, and *Cyclospora* were found in all types of leafy greens analyzed, while *Eimeria* was only absent in watercress. Other protozoa occurred at rates below 10%, and *Cystoisospora* was not detected in any samples. Helminths were present in almost half of the samples analyzed (49%), with *Strongylida* larvae accounting for 46% of them in all types of leafy greens, mainly in alfalfa (37.5%). Ancylostomidae eggs were absent (Figure 4).

## 4. Discussion

Foodborne diseases represent a major threat to global health, with enteroparasites being one of the most important. Protozoa and helminths commonly present in agricultural products due to fecal contamination cause serious gastrointestinal problems that, in cases of chronic infection, can lead to malnutrition and even death. Therefore, constant monitoring of agricultural foods is essential for the early detection of contamination and the prevention of diseases transmitted by their consumption. Regular surveillance and strict food safety measures are crucial to reduce the incidence of parasitic infections. This proactive approach is vital to protect public health and mitigate the risks and consequences of consuming contaminated food, particularly in regions with limited or no access to public health systems and services.

Government records indicate that 47.9% of Ecuador's rural population lives in poverty with an average monthly household income of $84.05, while 27.5% live in extreme poverty with an average income of $47.70. This is the case of the community in the province of Chimborazo, where this study was carried out, whose extremely poor population also has habits and customs inherited from their ancestors that may contribute to the lack of basic hygiene and sanitation measures. “Cities use septic tanks, while those closer to the capital have sewage systems; however, both discharge their wastewater into rivers and streams” [24].

There are few studies published on food safety in agricultural products from Ecuador, which is essential to know the rate of food contamination and understand its implications in parasitic transmission. Our study provides knowledge about the sanitary quality of agricultural products grown in the Andean region of Ecuador, an important production area that markets food at regional, national, and international levels. We found a high rate of overall parasitic contamination in products from the capital of Guano (74.5%), with 86.5% of leafy greens, 69.1% of vegetables, and 67.1% of fruits contaminated. This data are consistent with those reported by González-Ramírez et al. in the parish of San Andrés in Chimborazo, Ecuador, which revealed a slightly lower overall contamination rate (63.4%), with vegetables also showing the highest levels of contamination (76.9%) compared to fruits (48.4%) [2]. Moreover, protozoa were more prevalent than helminths in both regions, with *Blastocystis* and *Eimeria* being the most frequently identified parasites. In contrast, a study limited to the analysis of lettuce from the coastal region of Ecuador reported higher contamination frequencies (82.3%), with *E. nana* being the most frequently detected parasite in 35% of the samples [21].

The link between socioeconomic conditions and levels of contamination in agricultural products in the province of Chimborazo is evident. We believe that the high level of contamination of vegetables and fruits is due to the contamination of water in the region (100%), due to the lack of sanitary infrastructure, such as drinking water treatment plants, sewage systems, and wastewater treatment facilities, which together with the traditional agricultural practices of these indigenous populations, such as fertilizing crops with fresh animal manure, open defecation, breeding and living with parasitized animals, storing crops in unsanitary conditions, and selling products directly on the ground without sanitary controls or cleaning and disinfection processes in local markets, contribute to the dissemination and permanence of parasites. Contaminated water is used for the irrigation of crops, as well as for the preparation of solvents for pesticides and herbicides and the cleaning of tools used in planting, harvesting, and washing agricultural products [1, 2, 25–27].

When the results of our study are compared with some of the few works on parasitic contamination done in Ecuador, we find that the lettuces we collected in the high-altitude agricultural zone of the Ecuadorian Andes Sierra showed a higher frequency of parasites (93.3%) compared to lettuces from the Ecuadorian coast (82.3%), as reported by Bracho Mora et al. [21]. These authors also identified a high prevalence of *E. nana* (35.48%) and a low prevalence of *Blastocystis* sp. (6.45%), which contrast significantly with the 40% prevalence of *Blastocystis* sp. that we described in this study. The reason for this discrepancy is unknown, but we speculate that it could be attributed to differences in the origin of the samples: while the lettuce in our analysis was collected directly from the farms, the lettuce from the coast was purchased in markets, where it is typically cleaned and stripped of some of its outer leaves, which could reduce the parasitic load.

This proposal is supported by the fact that the prevalence of *Blastocystis* sp. in our study area (40.6%) is like that reported by Benites Salcedo, Castillo Valdivieso, and Jara Campos [28] in the Andean region of Peru (41.2%), reinforcing the influence of environmental, population, and cultural conditions shared between both countries [1]. Regarding global fruit contamination, the 67.1% detected in our study is comparable to the 70% reported in Brazil by Santos et al. [17]. However, in Brazil, geohelminths such as *A. lumbricoides* and hookworms were identified, whose prevalence is limited in our region due to altitude conditions that hinder their development. Conversely, Falcone et al. in Argentina reported a high prevalence of *Cryptosporidium* spp. (39.1%) and a low prevalence of hookworms (7.8%), *A. lumbricoides* (5.8%), and *Blastocystis* sp. (1.1%), highlighting the influence of altitude and other ecological factors on the regional variability in the distribution of parasitic species.

Parasitic contamination in Ecuadorian vegetables (69.1%) aligns with global ranges reported in Asia (47.5%–63%) and Africa (60%–62.3%) [13–15]. However, the comparison of helminth incidence is complicated because the microscopic analysis, used in this study, does not allow differentiation of *S. stercoralis* larvae from other free-living helminths, highlighting the need for more specific diagnostic approaches, such as immunological and molecular techniques, which are expensive. Where resources are limited, as in our case, a generalized report identifying them as *Strongylida* larvae is recommended. On the other hand, our results, together with others from Latin America and those from Asia and Africa, contrast with those from Europe, where parasitic prevalence is usually lower due to the disinfection of fruits and vegetables during packaging and prior to market distribution [7–9, 11, 12].

Comparing our results with those reported in meta-analyses on global contamination of fruits and vegetables, the global prevalence of protozoa in vegetables detected in Ecuador (69.1%) exceeds the global average of 20% (16%–24%) but falls within the range (6%–76%) found in the Southeast Asian WHO region [5]. The prevalence of *Blastocystis* sp. in our study (40.6%) is notably higher than that of *Cryptosporidium* sp. (5.4%), contrary to global findings, where the latter was the most prevalent protozoan parasite, reaching up to 15% [5]. Regarding helminth prevalence, contamination levels of Ecuadorian vegetables (28.7%) fall within the reported range (26%–37%), but Ecuadorian fruits show lower helminth contamination (9%) compared to the global mean of 20% (8%–37%) [6]. Interestingly, of the globally predominant helminth parasites such as *A. lumbricoides* (12%), *S. stercoralis* (12%), *T. trichiura* (9%), and *Toxocara* spp. (9%) [6], only *Strongylida* larvae (16%) were found in the Ecuadorian Andes.

Altitude-driven environmental conditions limit the development of most geohelminths, with free-living or veterinary-relevant *Strongylida* larvae predominating instead [1, 2]. The higher contamination of agricultural products from the Guano zone by protozoa (71.8%) compared to helminths (16.9%) correlates with our previous studies demonstrating the presence of protozoa (including *Blastocystis* sp., *Eimeria* sp., and *Entamoeba* sp.) in all analyzed water sources from the agricultural zone of the Ecuadorian Andes, compared to 45.9% contamination by helminths (*Dibothriocephalus* sp. and *Strongylida*) [25]. Similarly, the study of the excreta of herbivorous, omnivorous, carnivorous animals, birds, rodents, and leporids in the Guano capital found that they were predominantly parasitized (99.8%), with a higher rate of infection by protozoa (98.6%) than by helminths (21.4%) [26]. However, vegetables showed higher contamination with helminths than leafy greens, which may be attributed to the fact that carrots, radishes, and onions grow underground, where they come into direct contact with larvae and eggs of geohelminths. The risk arises from consuming these tubers raw, without adequate hygiene measures [15, 19]. The identification of *Blastocystis* sp. as the most frequent parasite in all the agricultural products analyzed suggests a high incidence in the population of the region.

*Blastocystis* is a ubiquitous parasite found in a variety of hosts, including humans, and is frequently detected in fecal samples around the world [16, 20]. This parasite is resistant to chlorination and can survive in diverse environmental conditions, making it a persistent contaminant [29]. However, the pathogenic potential of *Blastocystis* sp. is questionable, as some studies describe it as a human commensal while others suggest that certain subtypes of the parasite cause intestinal symptoms such as diarrhea, pain, and abdominal distension [30]. This dichotomy complicates public health interventions and underlines the need for detailed studies on the pathogenicity and epidemiology of *Blastocystis*. The risk of infection in children is of particular concern due to their increased vulnerability to intestinal infections and their propensity to eat contaminated foods [31, 32].

The predominance of protozoa over helminths in the region may also be influenced by the climate. The Andean region studied, located at 3900 m above sea level, experiences temperatures ranging from 5°C to 18°C that limit the viability of geohelminths, which require temperatures between 20°C and 30°C to become infectious. High levels of evapotranspiration and solar radiation in these Andean peaks reduce environmental humidity, further limiting the viability of geohelminths [25]. In addition, the surface layer of soils is composed of lithic materials of volcanic origin (Andosols, Inceptisols, and Histosols), which are very thin and do not allow helminth maturation, preventing worms from completing their biological cycle [33]. Only *Strongylida* larvae and Ancylostomidae eggs, likely from animals or free-living and not posing a risk to humans, were identified in the samples examined. In agreement, helminths causing disease in humans such as *A. lumbricoides*, *T. trichiura*, Ancylostomidae, and *S. stercoralis* have not been reported in coproparasitological analyses of individuals from the same canton [34]. Conversely, protozoa are not affected by climate and are highly represented [27].

One of the most important contributions of this study is that it reaffirms the propensity of green leafy vegetables to carry more contaminating parasites than other agricultural products. Among the factors that favor their contamination are irrigation with nonpotable water, direct contact of the leaves with contaminated soil, splashing of soil on the leaves during rain or irrigation, direct exposure to organic fertilizers prepared with contaminated water, the arrangement of the outer leaves that envelop the inner parts of the plant trapping soil and contaminants, and the irregularities and roughness of the surfaces that facilitate the adhesion of parasitic forms, so the ingestion of raw vegetable leaves in salads and sauces has been identified as the greatest risk [1, 16, 35]. Unlike green leafy vegetables, fruits and vegetables generally absorb water through their roots without contaminating the edible product [25]. This points to the need, at the consumer level, to thoroughly wash and disinfect edible leaves, which is a challenge since few people soak them in disinfectant solutions before consuming them [36]. At the producer level, measures such as watering and cleaning instruments with better quality water sources, avoiding the use of raw manure as fertilizer, promoting the application of heat-treated compost, covering the soil with plastic (plasticulture), and implementing cleaning and disinfection protocols at harvest, storage, and sales points should be taken to ensure a sanitary supply chain. To this end, local policies should be established to regulate markets and ensure that products sold comply with food safety standards [8, 9, 16, 34].

Finally, we consider that despite the limitation of this study on detailed identification of parasites, the findings of this research contribute significantly to the health sector by alerting about the levels of parasitic contamination in agricultural products harvested in the Ecuadorian Andean region, helping to explain the high prevalence of enteroparasites observed in human and animal populations in this area [1, 27, 34]. The results of the study serve as a critical call to action to improve public health interventions and agricultural practices. There is a critical need for targeted public health interventions, including education on proper food handling and hygiene, as well as measures to ensure the distribution of safe water and adequate excreta disposal to protect the population from infection.

## 5. Conclusions and Recommendations

This study highlights the significant parasitic contamination in agricultural products cultivated in the capital of Guano canton, Chimborazo Province, Ecuador. With a positivity rate of 74.5%, these foods present a substantial risk as carriers of infectious parasitic forms, particularly in leafy greens, which showed the highest contamination, followed by vegetables and fruits consumed raw. The higher prevalence of protozoa suggests that the extreme climatic conditions of the Andean mountains do not impede their survival and transmission, unlike the restrictions faced by helminths. The findings of this study not only underscore the urgent need to improve sanitation practices in agricultural environments to mitigate the health risks associated with parasitic infections but also provide a solid foundation for developing public policies and intervention strategies that ensure food safety, both locally and in the markets where these products are sold. Collaboration among authorities, communities, and public health experts is essential to effectively and sustainably address this issue.

## Figures and Tables

**Table 1:** Occurrence of parasites in agricultural products harvested in the capital of Guano canton.

**Parasites**	**Fruit**			**Vegetables**			**Leafy greens**			**Total**		
	**n** = 310			**n** = 188			**n** = 275			**n** = 773		
	**np**	**%**	**IC**	**np**	**%**	**IC**	**np**	**%**	**IC**	**np**	**%**	**IC**
*Blastocystis* sp.	135	43.5	(38–49)	67	35.6	(28.8–42.4)	112	40.7	(34.9–46.5)	314	40.6	(37.1–44.1)
*Eimeria* sp.	67	21.6	(17–26.2)	30	16	(10.8–21.2)	85	30.9	(25.4–36.4)	182	23.5	(20.5–26.5)
*Entamoeba* sp.	15	4.8	(2.4–7.2)	20	10.6	(6.2–15)	51	18.5	(13.9–23.1)	86	11.1	(8.9–13.3)
*Cryptosporidium* sp.	17	5.5	(3.0–8.0)	11	5.9	(2.5–9.3)	14	5.1	(2.5–7.7)	42	5.4	(3.8–7)
*Cyclospora* sp.	15	4.8	(2.4–7.2)	7	3.7	(1–6.4)	14	5.1	(2.5–7.7)	36	4.7	(3.2–6.2)
*Endolimax nana*	19	6.1	(3.4–8.8)	4	2.1	(0.1–4.1)	9	3.3	(1.2–5.4)	32	4.1	(2.7–5.5)
*Balantidium* sp.	1	0.3	(0–0.9)	0	0	0	19	6.9	(3.9–9.9)	20	2.6	(1.5–3.7)
*Chilomastix* sp.	2	0.6	(0–1.5)	7	3.7	(1–6.4)	1	0.4	(0–1.1)	10	1.3	(0.5–2.1)
*Cystoisospora* sp.	3	1.0	(0–2.1)	1	0.5	(0–1.5)	0	0	0	4	0.5	(0–1)
*Giardia* sp.	0	0	0	0	0	0	3	1.1	(0–2.3)	3	0.4	(0–0.8)
Total positives with protozoa	205	66.1	(60.8–71.4)	122	64.9	(58.1–71.7)	228	82.9	(78.4–87.4)	555	71.8	(68.6–75.0)
*Strongylida* larvae	27	8.7	(5.6–11.8)	53	28.2	(21.8–34.6)	46	16.7	(12.3–21.1)	126	16.3	(13.7–18.9)
*Passalurus ambiguus*	0	0	0	0	0	0	3	1.1	(0–2.3)	3	0.4	(0–0.8)
Ancylostomidae eggs	1	0.3	(0–0.9)	1	0.5	(0–1.5)	0	0	0	2	0.3	(0–0.7)
Total positives with helminths	28	9.0	(5.8–12.2)	54	28.7	(22.2–35.2)	49	17.8	(13.3–22.3)	131	16.9	(14.3–19.5)
Total positives with parasites	208	67.1	(61.9–72.3)	130	69.1	(62.5–75.7)	238	86.5	(82.5–90.5)	576	74.5	(71.4–77.6)

*Note:n* = sample size, np = samples positive for parasitic structures.

Abbreviation: IC = confidence interval.

## Data Availability

The datasets used and analyzed during the current study are available from the corresponding authors on reasonable request.

## References

[1] González-Ramírez L. C., Robalino-Flores X., De la Torre E. (2022). Influence of environmental pollution and living conditions on parasite transmission among indigenous Ecuadorians. *International Journal of Environmental Research and Public Health*.

[2] González-Ramírez L. C., Djabayan-Djibeyan P., Prato J. G. (2023). Field study of parasitic contamination of fruits, vegetables and leafy greens in the Ecuadorian Andes. *F1000Research*.

[3] WHO (2017). Progress on Drinking Water, Sanitation and Hygiene. https://washdata.org/sites/default/files/documents/reports/2018-01/JMP-2017-report-final-highlights.pdf.

[4] Davies O. L. (2015). *WHO’s first ever global estimates of foodborne diseases find children under 5 account for almost one third of deaths*.

[5] Badri M., Olfatifar M., Karim M. R. (2022). Global prevalence of intestinal protozoan contamination in vegetables and fruits: a systematic review and meta-analysis. *Food Control*.

[6] Eslahi A. V., Olfatifar M., Karim M. R. (2022). Global incidence of helminthic contamination of vegetables, cucurbits and fruits: a systematic review and meta-analysis. *Food Control*.

[7] Caradonna T., Marangi M., Del Chierico F. (2017). Detection and prevalence of protozoan parasites in ready-to-eat packaged salads on sale in Italy. *Food Microbiology*.

[8] Barlaam A., Temesgen T. T., Tysnes K. R. (2021). Contamination of fresh produce sold on the Italian market with *Cyclospora cayetanensis* and *Echinococcus multilocularis*. *Food Microbiology*.

[9] Barlaam A., Sannella A. R., Ferrari N. (2022). Ready-to-eat salads and berry fruits purchased in Italy contaminated by *Cryptosporidium* spp., *Giardia duodenalis*, and *Entamoeba histolytica*. *International Journal of Food Microbiology*.

[10] Trelis M., Sáez-Durán S., Puchades P. (2022). Survey of the occurrence of *Giardia duodenalis* cysts and *Cryptosporidium* spp. oocysts in green leafy vegetables marketed in the city of Valencia (Spain). *International Journal of Food Microbiology*.

[11] Moreno-Mesonero L., Soler L., Amorós I., Moreno Y., Ferrús M. A., Alonso J. L. (2023). Protozoan parasites and free-living amoebae contamination in organic leafy green vegetables and strawberries from Spain. *Food and Waterborne Parasitology*.

[12] Healy S. R., Morgan E. R., Prada J. M., Betson M. (2023). From fox to fork? *Toxocara* contamination of spinach grown in the south of England, UK. *Parasites and Vectors*.

[13] Mufida D. C., Armiyanti Y., Putri E. R. (2022). Bacterial and parasitic contamination of raw vegetable: potential risk for food-borne diseases. *International Journal of Public Health Science (IJPHS)*.

[14] AL-Qorom S. M., Abu-Sharar T. M., Albdaiwi R. N. (2023). Parasitic contamination of soil and vegetable crops irrigated with raw wastewater: a case study from Al-Farâa, Palestine. *International Journal of Recycling of Organic Waste in Agriculture*.

[15] Yahia S. H., Etewa S. E., al Hoot A. A. A. (2023). Investigating the occurrence of soil-transmitted parasites contaminating soil, vegetables, and green fodder in the east of Nile Delta, Egypt. *Journal of Parasitology Research*.

[16] Falcone A. C., Zonta M. L., Unzaga J. M., Navone G. T. (2023). Agricultural practices and intestinal parasites: a study of socioenvironmental risk factors associated with leafy vegetable production in La Plata horticultural area, Argentina. *Parasite Epidemiology and Control*.

[17] Santos V. H., Borges J. M. P., Santos K. S. (2019). Study of the prevalence of helminths and protozoa in fruits marketed in street markets in a city inside of Bahia. *International Journal of Advanced Engineering Research and Science*.

[18] Polo G., Benavides C. J., Astaiza J. M., Vallejo D. A., Betancourt P. (2016). Enteroparasite determination in *Lactuca sativa* from farms dedicated to its production in Pasto, Colombia. *Biomédica*.

[19] Puig Peña Y., Leyva Castillo V., Rodríguez Suárez A. (2014). Calidad microbiológica de las hortalizas y factores asociados a la contaminación en áreas de cultivo en La Habana. *Revista Habanera de Ciencias Médicas*.

[20] Pérez-Cordón G., Rosales M. J., Valdez R. A., Vargas-Vásquez F., Cordova O. (2008). Detection of water-borne and food-borne intestinal parasites of Trujillo, Peru. *Revista Peruana de Medicina Experimental y Salud Publica*.

[21] Bracho-Mora A. M., Loor-Bravo E. Z., Nevarez-Zevallos G. R., Rivero de Rodríguez Z., Arteaga-Quiroz M. A. (2022). Determinación de parásitos intestinales en *Lactuca sativa*, expendidas en el mercado central de Portoviejo, Manabí-Ecuador. *Kasmera*.

[22] Rivero de Rodríguez Z., Fonseca R., Moreno Y., Oroño I., Urdaneta M. (1998). Detección de parásitos en lechugas distribuidas en mercados populares del Municipio Maracaibo. *Kasmera*.

[23] Garcia L. S., Bruckner D. A., Brewer T. C., Shimizu R. Y. (1983). Techniques for the recovery and identification of *Cryptosporidium* oocysts from stool specimens. *Journal of Clinical Microbiology*.

[24] Instituto Nacional de Estadísticas de Ecuador (2020). INEC. https://www.ecuadorencifras.gob.ec/institucional/home/.

[25] González-Ramírez L. C., Falconí-Ontaneda F. A., Yaucén-Rodríguez M. (2020). Dispersión hídrica de enteroparásitos en una zona agropecuaria de gran altitud, en Los Andes Ecuatorianos. *Kasmera*.

[26] Quispe Monar S. D., Caiza Reinoso D. E. (2020). *Identificación de enteroparásitos en animales que actúan como reservorios en Pungal Grande y San Pedro, Cantón Guano, Chimborazo*.

[27] González-Ramírez L. C., Vázquez C. J., Chimbaina M. B. (2021). Ocurrence of enteroparasites with zoonotic potential in animals of the rural area of San Andres, Chimborazo, Ecuador. *Veterinary Parasitology: Regional Studies and Reports*.

[28] Salcedo D. B., Valdivieso C. C., Campos C. J. (2019). Contaminación parasítica de hortalizas de consumo humano expendidas en mercados de Trujillo, Perú. *REBIOL*.

[29] Mokhtar A. B., Karanis P., Schou C., Ahmed S. A. (2023). The impact of chlorine, ultraviolet-C, and microwave treatment on the survivability of *Blastocystis* sp. cysts. *Journal of Water & Health*.

[30] Rudzińska M., Sikorska K. (2023). Epidemiology of *Blastocystis* infection: a review of data from Poland in relation to other reports. *Pathogens*.

[31] Matovelle C., Tejedor M. T., Monteagudo L. V., Beltrán A., Quílez J. (2022). Prevalence and associated factors of *Blastocystis* sp. infection in patients with gastrointestinal symptoms in Spain: a case-control study. *Tropical Medicine and Infectious Disease*.

[32] Nithyamathi K., Chandramathi S., Kumar S. (2016). Predominance of *Blastocystis* sp. infection among school children in peninsular Malaysia. *PLoS One*.

[33] Ayala-Izurieta J. E., Márquez C., García V., Recalde-Moreno C., Rodríguez-Llerena M., Damián-Carrión D. (2017). Land cover classification in an Ecuadorian mountain geosystem using a random forest classifier, spectral vegetation indices, and ancillary geographic data. *Geosciences*.

[34] González Ramírez L. C., Proaño Valverde J. M., Silva Durán N. E., Orozco Pilco J. A. (2023). Enteroparasitosis: un problema sanitario en residentes de la zona montañosa de Ecuador. *Anatomía Digital*.

[35] Temesgen T. T., Stigum V. M., Robertson L. J. (2022). Surveillance of berries sold on the Norwegian market for parasite contamination using molecular methods. *Food Microbiology*.

[36] Ramos B., Miller F. A., Brandão T. R. S., Teixeira P., Silva C. L. M. (2013). Fresh fruits and vegetables - an overview on applied methodologies to improve its quality and safety. *Innovative Food Science & Emerging Technologies*.

